# Does Nutrition Knowledge and Practice of Athletes Translate to Enhanced Athletic Performance? Cross-Sectional Study Amongst Nigerian Undergraduate Athletes

**DOI:** 10.5539/gjhs.v7n5p215

**Published:** 2015-03-16

**Authors:** Oluyemisi F. Folasire, Abiola A. Akomolafe, Rasaki A. Sanusi

**Affiliations:** 1 Department of Human Nutrition, College of Medicine, University of Ibadan, Nigeria & Department of Family Medicine, University College Hospital, Ibadan, Nigeria; 2 Department of Human Nutrition, College of Medicine, University of Ibadan, Nigeria

**Keywords:** athletic performance, bioelectrical impedance analysis, body composition, dietary pattern, hand dynamometer, nutrition knowledge

## Abstract

**Introduction and Objectives:**

Nutrition knowledge of an athlete, as well as practice, is expected to influence athlete’s performance. The study assessed the nutrition knowledge and practice as well as athletes’ performance and identified the factors predicting the athletes’ performance.

**Methodology:**

A cross-sectional survey, involved 110 purposively selected undergraduate athletes (47 females, 63 males) of University of Ibadan, Nigeria, between July 2013 and December 2013. A semi-structured, self-administered questionnaire assessed the nutrition knowledge and practice. 24-hr diet recall and food frequency questionnaire were done. Anthropometric measurements were taken; body composition was determined by bioelectrical impedance analysis method. Handgrip strength (HGS), as an indirect measure of athlete performance, was assessed with the hand dynamometer. Chi-square and t-test analysis were used for the bivariate analysis. Pearson correlation and simple linear regression were used to determine relationships and predict athletic performance. The level of statistical significance was p<0.05.

**Results:**

More than half (58.2%) had good nutrition knowledge (NK), and 62.7% had good nutrition practices (NP). Majority (75.4%) had normal handgrip strength (HGS). More than 70.0% frequently do not consume cereals, roots and tubers, fruits and vegetables, legumes/nuts. About 30.0-40.0% frequently do not consume eggs/milk, meat/fish. Having good NK was significantly associated with good NP (χ^2^ = 15.520, p=0.000), but not with athlete’s performance (HGS). There is no significant correlation between NK, NP, and HGS. There is a significant positive correlation between HGS and lean muscle mass (LMM) (r=.670, p=0.000), weight (r=.492, p=0.000), height (r=.521, p=0.000) and energy intake (r=.386, p=0.000). There is a significant negative correlation between HGS and percentage body fat (r=-.400, p=0.000). Athletes’ performance was significantly predicted by the resting metabolic rate (β= .454 C.I=0.011 to 0.045, p=0.003), Lean muscle mass (β =.297 C.I=.059 to 0.562, p=0.024) and the weight (β =.228, C.I=1.852 to .489, p=0.047).

**Conclusion:**

Having good nutrition knowledge or practice did not directly determine athletic performance. However, there is the need for nutrition education interventions, to improve athlete’s performance by promoting adequate energy intake, lean muscle mass and appropriate weight gain in athletes.

## 1. Introduction

Athletic performance can be enhanced by consumption of adequate nutrition. Nutritional requirement in an active individual is dependent on the extent of activity performed. Most athletes understand that proper fuelling through optimal nutrition with adequate nutrition knowledge is an essential and integral part of a training program (Rosenbloom, Jonnalagadda & Skinner, 2006). However, most athletes remain poorly educated about healthy nutritional practices and are untrained in making appropriate daily nutritional choices (Hornstrom, Friesen, Ellery, & Pike, 2011; Schmalz, 1993; Jacobson, Bert, & Gemmell, 1991). Majority of athletes still do not translate the knowledge of adequate nutrition into appropriate dietary choices (Sakamaki, Toyama, Amamoto, Liu, & Shinfuku, 2005). Nutrition knowledge is an important factor influencing dietary habits and food choices and thus, crucial in sports nutrition (Cupisti, Alessandro, Castrogiovanni, Barale, & Morelli, 2002).

Current knowledge about this issue is that the impact of nutrition knowledge of athletes on their dietary intake is equivocal. A recent systematic review reported a weak positive correlation between nutrition knowledge and dietary intakes of athletes (Heaney, O’Connor, Michael, Gifford, & Naughton, 2011). Moreover, a significant increase in total energy, carbohydrates, and protein intakes, as well as increased nutrition knowledge was reported in another study (Valliant, Emplaincourt, & Wenzel, 2012; Heaney et al., 2011).

Impaction of proper nutrition knowledge to athletes’ at the tertiary levels of education has been shown to improve with nutrition intervention. Nutrition education interventions have an effect on eating habits and dietary practices (Sakamaki et al., 2005; Zawila, Steib, & Hoogenboom, 2003). However, only a few athletes’ reflected the concept of adequate nutrition when selecting from a food menu (Sakamaki et al., 2005). High level of nutrition knowledge has been reported to be related to positive dietary habits (Sakamaki et al., 2005). The source of nutrition information amongst athletes is another factor that determines their nutrition knowledge. Most studies report Coaches, rather than Nutritionists/Dietitians as the source of nutrition information for athletes. Moreover, the Coaches may not be suitably qualified to provide such services (Jessri, Rashidkhani, & Zinn, 2010; Arnheim & Prentice, 2000). Nutrition knowledge of athletes has been shown to correlate positively with practice (Hornstrom et al., 2011; Cupisti et al., 2002; Jessri et al., 2010). However, no significant correlation was reported between knowledge and practice (Supriya, 2013). Sports education students and teachers were reported not to pay importance to their diet and do not know the significance of nutrition in sports performance (Ozdogan & Ozcelik, 2011). Athletes that realize the crucial role of an adequate diet and reflect the knowledge in their behaviors and dietary practice are more successful in sports life (Ozdogan & Ozcelik, 2011; Frederick & Hawkins, 1992). It is, however, important to note that nutrition knowledge does not always translate to dietary practice (Cupisti et al., 2002). Opportunity for impacting adequate and proper nutrition education to athletic team; coaches and trainers of athletes will improve performance of athletes (Torres-mcgehee, Pritchett, Zippel, Minton, Cellamare, & Sibilia, 2012).

For optimal performance, nutritional needs for peak athletic performance must be met, and includes sufficient energy intake, adequate hydration, and timing of meals. Student athletes and their advisers/coaches often mislead or have an erroneous belief about the requirements in sports nutrition (Sakamaki et al., 2005; Zawila et al., 2003; Nancy, Connie, Vickery, & Mcbee, 2005). The recommended adequate diets, for an athlete, must involve at least three meals per day, in which the daily protein content must be 10-15% of the total energy, carbohydrate content is 55.0%, and fat consumption is 30% or less (Fahey, Insel, & Roth, 2001; Brooks, Fahey, White, & Baldwin, 2000). Proper nutrient intake corresponds to peak athletes’ functionality, and nutrient insufficiency may lead to reduced athletic performance (Hornstrom, 2011; Potgieter, 2013). Restriction of energy intake and loss of muscle and fat mass negatively affects athletic performance (A.D.A. & D of Canada, 2009). It is important for athletes to maintain appropriate fat mass and weight for optimal performance. The objectives of the current study were to assess the nutrition knowledge, nutrition practice and athletes’ performance and identify the factors predicting the athletes’ performance.

## 2. Materials and Methods

### 2.1 Study Design

A cross-sectional study was conducted among 110 undergraduates of the University of Ibadan, Nigeria between July, 2013 and December, 2013.

### 2.2 Study Site and Participants

The study was carried out at the various training grounds at the campus of the University of Ibadan, Nigeria. The University of Ibadan is the premier tertiary Institution in Nigeria. There are 15 sports facilities at the University campus. Some training grounds in the University are the basketball court, football field, swimming pool, an old gymnasium, squash court, volleyball court.

### 2.3 Sampling Technique

Purposive sampling was used to select all consenting athlete (N=110) to participated in the study. Inclusion criteria; being undergraduate athletes that actively engage in at least a sport. Respondents that reported been ill two weeks before the study were excluded.

### 2.4 Data Collection Procedures

Using a self- administered, semi-structured questionnaire, information on socio-demographic data, nutrition knowledge, and practice were collected. Information on the participants’ 24-hour diet recall, food frequency questionnaire (FFQ), and anthropometric measurements were documented. The FFQ was used to determine the dietary pattern. In order to reduce inaccuracy in nutrient intakes, 3 repeated consecutive dietary recalls were done. Trained field workers used food models to assist the respondents in estimating portion sizes. The anthropometric measurements including weight, height taken according to standardized procedures (Cupisti et al., 2002), (Lohman, Roche, & Martorell, 1988). Weight (to nearest 0.1kg) and body composition, resting metabolism were taken using a digital Omron body composition analyzer, model BF 400, height (to nearest 0.1m) with Scales 2000 stadiometer. The handgrip strength (HGS), as an indirect measure of athletes’ performance, was assessed with the hand dynamometer (Gibson, 2005). All measurements were performed twice, and average values documented. The Body mass index (BMI) was calculated using weight (kg) divided by height squared (m^2^) and categorized using the World Health Organization (WHO) cut-off points.(W.H.O., 2011; Mohsin et al., 2010). The questions on nutrition knowledge and practice were structured using previous literatures (Zawila et al., 2003; Supriya, 2013). To assess the athletes’ knowledge of food nutrients, common sources of nutrients and their functions. The questionnaire was face and content validated by nutrition lecturers and dietitians. It was checked immediately after filling to minimize missing data. The questionnaire was pretested in a similar group of athletes at another tertiary institution, with good internal consistency, Cronbach’s alpha of 0.75.

### 2.5 Data Analysis

All statistical analyzes were performed using the Statistical Package for the Social Sciences (SPSS, IL version 20.0). Mean nutrition knowledge (NK) score was calculated based on the responses of the participants. The participants scored 1, for every correct answer and zero, for every wrong answer. Those that chose ‘not sure option’ scored zero too, the scores were added, and the mean nutrition knowledge score was 9.00 ± 2.53. Those that scored less than the mean score were classified as having a poor knowledge while those that scored more than the mean score were classified as having good NK. The mean nutrition practice (NP) score was calculated based on the responses of the participants. The participants scored 1, for every correct answer and scored zero for every wrong answer. Those that chose ‘unsure option’ scored zero too. The scores were added and gave mean nutrition practice score of 8.00 ± 2.55. Scores less than the mean score are classified as poor NP, while, while, scores more than the mean score as good NP.

The adapted total dietary assessment (TDA) was used to determine the mean nutrient intakes of the athletes from the repeated 24-hour dietary recall, to reduce under and over-reporting of energy intakes (Sanusi & Falana, 2007). The Food Frequency Questionnaire was used to determine the frequency of consumption from food groups. Categorical variables were compared with the chi-square test, mean NK and NP scores with the student t-test. Correlation and simple linear regression analysis were the inferential statistical test that was also done. With the enter mode function, predicting relationship between independent variables (anthropometric, nutrition knowledge and dietary practice scores, selected mean nutrients intake) and the dependent variable (hand grip strength), was determined. The level of statistical significance was set at p<0.05.

Quality control: Study protocol was pre-tested, among athletes in another tertiary institution. The field workers were trained and re-trained halfway into data collection process. Each questionnaire was checked immediately to minimize missing data. New batteries were available for the Omron body composition scale and removed at the end of each day.

### 2.6 Ethical Clearance

The study protocol was approved by the Institutional Review Committee of the University College Hospital/University of Ibadan (UI/UCH) ethical board.

## 3. Results

Out of the 110 undergraduate athletes recruited, 57.3% were male, and 42.7% were female. About 39.0% of the respondents were in their semi-final and final year while 60.9% were in first to third year of study. About half (50.9%) of the respondents play ball games; 12.7% and 17.3% play racket and combat respectively, and 19.1% swim. These games were done daily or at least 3 times weekly by 25.5% respectively. Seventy-eight percent responded that they did not seek nutritional advice while 21.8% respondents sought nutritional advice. Of those that sought nutrition advice, 41.7% were advised by coaches, 37.5% by nutritionist/dietitians, 8.3%, and 12.5% talk to friends and medical doctors/nurses about nutrition. Majority (83.3%) often took to the specialist’s opinion, while 16.7% do not. Eighty percent of the selected athletes were in the age group 20-24 years and 12.7% and 7.3% belong to age groups 25 and above and less than 20 years respectively. The mean age was 22.0±2.39 years and is reported with the anthropometric characteristic in Table 1.

**Table 1:** Anthropometric characteristics of respondents

Variable	Mean(SD)	Minimum	Maximum
Mean age (years)	22.06(2.4)	17.0	32.0
Weight (kg)	68.66(8.8)	51.1	93.5
Height (m)	1.71(0.1)	1.6	2.0
BMI (kg/m^2^)	23.32(2.6)	19.1	30.4
Waist circum.(cm)	81.83(6.1)	68.0	99.0
Hip circum.(cm)	97.52(7.6)	78.5	114.8
Body fat (%)	26.12(8.3)	6.9	47.3
Muscle mass (kg)	37.10(9.1)	22.0	79.9
Visceral fat (%)	4.21(1.8)	1.0	10.0
Resting metabolism (Kcal)	1553(155.3)	1244.0	1917.0
Right-hand grip strength(kg)	38.33(9.5)	20.1	58.2
Left-hand grip strength (kg)	35.05(9.5)	17.6	58.9

More than half (n=64, 58.2%) of the athletes had good nutrition knowledge scores, while 46 (41.8%) had poor nutrition knowledge scores. Almost all the respondents (90.9%) responded that food should be eaten at least three times daily, 64% of the athletes responded correctly that there are 6 classes of nutrients, 69.1% answered correctly that foods rich in carbohydrates are the main sources of energy in the body. More than half (57.3%) of the athletes answered correctly that their food intake should increase since they are athletes. A high percentage (63.8%) responded correctly to whether male and female athletes use the same amount of energy during exercise. Of all the athletes surveyed, 59.1% responded correctly that sport drinks are the best to replace fluids lost on the field of play, and 55.5% wrongly responded that vitamins are good sources of energy.

**Table 2:** Nutrition knowledge of the athletes

S/N	Nutrition Knowledge questions	Correct Response		Incorrect Response	

		n	%	n	%
1.	How many times should we eat in a day?	100	90.9	10	9.1
2.	How many classes of nutrients do we have?	70	63.6	40	36.4
3.	Do you think foods rich in carbohydrates are the main sources of energy in the body?	76	69.1	34	30.9
4.	As an athlete, my food intake should increase	63	57.3	47	42.7
5.	Can lack of iron in the diet result in fatigue, injury and illness?	70	63.6	40	36.4
6.	Do you think sports drinks are the best to replace body fluids lost on the field of play?	65	59.1	45	40.9
7.	Are vitamins good sources of energy?	19	17.3	91	82.7
8.	Alcohol consumption can negatively affect the absorption and utilization of nutrients?	77	70.0	33	30.0
9.	Eating of snacks is as good as eating home prepared foods?	98	89.1	12	10.9
10.	Do you think foods rich in sugar, jam and honey are suitable sources of energy for athletes?	35	31.8	75	68.2
11.	The last meal before a competition should be consumed at least 3 hours before a competition	91	82.7	19	17.3
12.	Males and females of the same group use up the same amount of energy during exercise	75	68.2	35	31.8
13	Do you think fruits and vegetables are important sources of vitamins and minerals?	91	82.7	19	17.3
14	Do you think milk and milk products are good sources of calcium?	82	74.5	28	25.5

**Figure 1:**
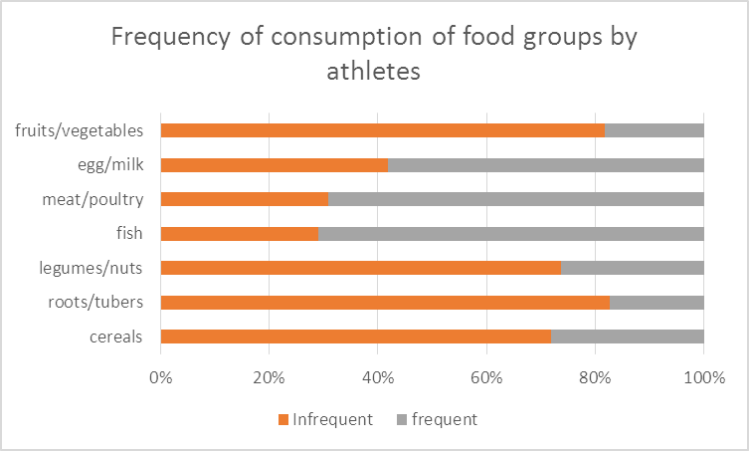
Consumption pattern from food groups by athletes

More than half of the athletes did not meet their recommended dietary allowance for the macronutrients (50.9% for carbohydrate, 65.5% for protein and 94.5% for fat). Also, more than half had inadequate intake of some vitamins and minerals (80.9% for vitamin C, 84.5% for vitamin B12, 99.1% for calcium, 76.4% for sodium, 95.5% for potassium, 72.7% for magnesium), though 80% had excess intake of iron. The food groups frequently consumed amongst majority of the athletes were fish, poultry, egg/milk. The reported usual dietary pattern was different from what they actually consumed, as shown in Table 3 and Figure 1.

**Table 3:** Nutrition practice of the athletes

	Nutrition practice questions	Yes		No	

		n	%	n	%
1.	Do you use supplements like multivitamin as an athlete?	22	20.0	88	80.0		
2.	I consume lots of fruits and vegetables	81	73.6	29	26.4		
3.	I skip meals before a competition or an event	45	40.9	65	50.1		
4.	I eat just before an event	25	22.7	85	77.3		
5.	I eat just after an event	47	42.7	63	57.3		
6.	I consume sports drinks every day during practice or when I feel dehydrated	36	32.7	74	67.3		
7.	I eat adequate diet daily	58	52.7	52	47.3		
8.	I change my pattern of eating at the time of a competition	59	53.6	51	46.4		
9.	I always take my breakfast daily	54	49.1	56	50.9		
10.	I consume lots of water during and after training/competition	92	83.6	18	16.4		
11.	I always eat at least one hour before training/competition	49	44.5	61	55.5		
12.	I prefer snacks to special diet before training and competition	31	28.2	79	71.8		
13.	I eat at least 3 times daily	49	44.5	61	55.5		
14.	I consume milk and milk products daily	35	31.8	75	68.2		
15.	I consume alcohol to enhance my performance	13	11.8	97	88.2		

More than 70.0% frequently do not consume cereals, roots and tubers and another above 70.0% frequently do not consume fruits and vegetables, legumes/nuts. Only about 30.0-40.0% frequently do not consume eggs/milk, meat/fish, Figure 1.

Majority of the athletes that had good nutrition knowledge scores also had good nutrition practice scores, also majority that had poor nutrition knowledge scores also had poor practice scores, which is statistically significant (χ^2^ =15.520, p=0.000), Table 4.

**Table 4:** Nutrition knowledge and nutrition practice of athletes

		Nutrition Practice Scores			

		Poor	Good	χ^2^	P
**Nutrition Knowledge Scores**	Poor	27	19	15.520	0.000*
	Good	14	50		
	Total	41	69		

* Statistically significant p<0.05.

There is no significant correlation between nutrition knowledge and anthropometric parameters of the respondents but the nutrition practice scores of the respondents negatively correlated with their HC and VF (r= -.208, -.218 respectively, p<0.05). There is a significant positive correlation between the NK and the NP scores. There is also moderate to strong positive correlation between hand grip strength (HGS), the measure of athletic performance and most anthropometric parameters; weight (r=.492), height (r=.521), LMM (r=.670), RM (r=.757), p<0.05 respectively. The HGS is negatively correlated with the percentage body fat (r=-.400, p<0.05), but positively with energy intake (r=.266, p<0.05), There is significant positive correlations between iron intakes and LMM (r=.241), Energy intake (r=.309) and Calcium intake (r=.693, p<0.05) and negatively with BMI (r-.190) and percentage body fat (r=-.208) p<0.05 respectively (Table 5).

**Table 5:** Correlation analysis of athletes’ selected anthropometric and nutritional parameters and selected nutrient intakes

	Weight	Height	BMI	WC	HC	%BF	LMM	VF	RM	HGS	NK	NP	Ene.I	Calc.I	Iron.I
Weight(kg)	1							
Height(m2)	.580*	1							
BMI(kg/m2)	.718*	-.317	1						
WC(cm)	.457*	.113	.431*	1						
HC(cm)	.456*	-.012	.568*	.724	1					
%BF	.186	-.310*	-.487*	.340	.680*	1					
LMM (kg)	.194	.450*	-.141	-.106	-.420*	-.729*	1				
VF (%)	.535*	-.030	.645*	.417*	.476*	.367*	-.134	1				
RM(kcal)	.633*	.601*	.259*	.270*	-.035	-.429*	.717*	.228	1			
HGS(kg)	.492*	.521*	.159	.106	-.118	-.400*	.670*	.054	.757*	1			
NK score	-.005	.097	-.091	.058	-.001	-.176	.162	-.036	.058	.113	1		
NP score	-.027	.155	-.157	-.092	-.208*	-.165	.157	-.218*	.055	.157	.396*	1		
Energy Intake(kcal)	.122	.178	.026	-.041	-.110	-.221*	-.323*	-.007	.298*	.386*	.180	.266*	1	
Calc. Intake(mg)	.055	.107	-.007	.012	-.021	-.141	.169	.012	.145	.180	-.231*	-.102	.265*	1	
Iron Intake (mg)	-.055	.176	-.190*	-.035	-.084	-.208*	.241*	-.101	.150	.170	-.136	.097	.309*	.693*	1

* Significant at level <0.05.

Abbreviations: BMI, body mass index; WC, waist circumference; HC, hip circumference; %BF, percentage body fat; LMM, lean muscle mass; VF, visceral fat; RM, resting metabolism; HGS, hand grip strength (non-dominant hand);NK, nutrition knowledge; NP, nutrition practice, kcal; kilocalories, mg; milligrams, Ene.I;energy intake, Calc.I; calcium intake, Iron I; iron intake.

The best predictor of athlete’s performance was the resting metabolism (β=.428, 95% C.I. =1.852 to 0.043, p=0.003), lean muscle mass (β = .274, 95% C.I.= .809 to .535, p=0.024) and the weight (β =.228, 95% C.I.= 1.852 to .489, p=0.047). About 61% of the variance in the handgrip strength, which is our measure of athletic performance can be explained by this model, Table 6.

**Table 6:** Linear regression analysis

Variable	β	t	95% C.I	P value
Weight	.228	2.011	1.852 to 0.489	.047*
WC	-.019	-.195	0.809 to 0.278	.846
HC	-.046	-.357	-0.423 to 0.265	.722
%BF	.064	.524	1.852 to 0.352	.601
LMM	.274	2.287	0.809 to 0.535	.024*
VF %	-.117	-1.505	-0.423 to .197	.135
RM	.428	3.059	1.852 to .043	.003*
NK	.027	.374	-.437 to .640	.710
NP	.036	.498	-.401 to .669	.620
Energy intake	.128	1.852	.000 to .003	.067
Calcium intake	.069	.809	-.003 to .007	.420
Iron intake	-.037	-.423	-.071 to .046	.673

Adjusted R^2^= .613, F= 15.397, p=0.000;

* Significant at level < 0.05.

## 4. Discussion

The present study investigated the relationship between nutrition knowledge, dietary practice and athletic performance. The consideration that, inadequate knowledge of nutrition, and not been aware of additional needs for nutrients was associated with suboptimal athletic performance reported by Ozdogan and Ozcelik was confirmed in the current study (Ozdogan & Ozcelik, 2011). Nutrition knowledge (NK) and nutrition practice (NP) do not necessarily translate to athlete’s performance as there was no significant correlation between NK, NP and handgrip strength (HGS). The hand grip strength was the measure of athletic performance in the study.

Current study was in agreement with a previous finding of a significant correlation between NK and NP scores of athletes (Hornstrom et al., 2011), but in contrast to Supriya’s report (Supriya, 2013). However, in agreement with Heaney et al. (2011), there is a weak positive correlation between nutrition knowledge and energy intake in this study (Heaney et al., 2011). With regards to response to nutrition knowledge questions, a higher proportion compared to previous study correctly responded “lack of iron can result in fatigue and injury”, and similar proportion correctly respond “food intake of an athlete is more than normal population” (Nazni &Vimala, 2010; Supriya, 2013). In addition, 82.7% of the participants (82.7%) correctly responded to the statement “are vitamins good sources of energy” as false, similar to the previous study (Ozdogan & Ozcelik, 2011)

Compared to Nazni and Vimala (2010) report, higher percentage (40.9%) in current study skips meals before a competition, but lower percentage, (53.5%) of athletes responded to changing their diet before a competition. This finding is however, contrary to a recent report from the same environment by Oladunmi and Sanusi (2013), were lower percentage (33.0%) consume sports drink during practice and another forty percent skip meals at time of competition (Oladunmi & Sanusi, 2013).

Also, the finding of no significant correlation between the energy intake and weight, was contrary to previous work (Nazni & Vimala, 2010). More than half of the respondents, did not meet the macronutrients RDA for athletes, and of some micronutrient, including vitamin B12, vitamin C and calcium similar to previous reports (Cupisti et al., 2002; Nazni & Vimala, 2010; Valliant et al., 2012). However, this is in contrast to previous study reporting intake of a large amount of carbohydrate-rich fluids and vitamin and mineral supplements (Saris et al., 1989). Food groups frequently consumed by the athletes are eggs, milk, meat and fish, while about 70% less frequently consume healthy roots and tubers, legumes, fruits and vegetables, similarly reported (Oladunmi & Sanusi, 2013) Also, the inadequacy of respondents’ energy intake, highlighted the unhealthy eating and food group consumption pattern of the athletes. This may invariably reduce performance. It is recommended that all athletes should consume diets that provide the minimal RDA for all micronutrients, and this is guaranteed if the energy requirements are met (American Dietetic Association & Dietitians of Canada, 2009; ADA, 2000; Potgieter, 2013).

In the agreement with Hornstom et al. (2011), only 21.8% sought nutrition advice and just 37.5% have access to such information from dietitians/nutritionist (Hornstrom et al., 2011). A finding consistent with previous reports is that, the athletes’ preferred to access nutrition information from athletic trainers rather than dietitians (Jacobson, & Gemmell, 1991a; Jacobson, Sobonya, & Ransone, 2001b; Burns, Schiller, Merrick, & Wolf, 2004; Froiland, Koszewski, Hingst, & Kopecky, 2004). This may also have been responsible for the majority of the athletes not frequently consuming the common healthy staples in Nigeria (roots and tubers) and might have translated in inadequate energy intake, similar to a previous report (Hoogenboom, Morris, Morris, & Schaefer, 2009). Further emphasizing the importance of coaches and athlete trainers having adequate nutrition information, which equips the team to be abe to guide athletes better (Torres-mcgehee et al., 2012).

The current study also revealed athletes that about 70% were not frequently consuming cereals, roots, and tubers, the main energy source of carbohydrate in Nigeria and were not meeting the RDA for macronutrients, similar to a prevous report (Hoogenboom et al., 2009). Though, the article did not describe the adequacy of nutrient intakes per se, however, a strong positive correlation was reported between the hand grip strength and energy intake. Thus, confirming the need for adequate energy intake for optimal athletic performance (ADA, 2000; American Dietetic Association & Dietitians of Canada, 2009). In addition, some misconceptions about the dietary practice were identified such as more than fifty percent of the respondents change their diet at time of competitions which may invariably impair athletes’ performance.

The study finding that the athletes’ performance is best predicted by the resting metabolism, a component of the energy requirement explains the indirect role that nutrition knowledge or practice play. Though there is minimal work in this environment to discuss the predictor of athletes’ performance. The general option holds that inadequate energy intake prevents adequate intake of micronutrients, and performance ultimately will be impaired (Hornstrom et al., 2011). Despite, the moderate but positive correlation between energy intake and hand grip strength, it is not a significant predictor. The energy intake is also positively correlated with the resting metabolism that emerged as the best predictor of the hand grip strength, our measure of athletic performance. Another predictor of the athletic performance was the lean muscle mass, which has a strong positive correlation with the resting metabolism, hand grip strength and iron intake, but is negatively correlated with energy intake. The muscle mass is determined by the protein status of an individual, and for activity of the muscle cell, iron is very important (ADA, 2000; American Dietetic Association & Dietitians of Canada, 2009). About 61% of the variance in the handgrip strength of the respondents can be explained by the model, which is strength of the study.

## 5. Conclusion

Nutrition knowledge and practice do not directly determine athletes’ performance, rather when nutrition knowledge and practice is channeled to meeting energy requirements, ensuring appropriate muscle mass and weight, then can enhanced athletic performance be expected. Athletes cannot sustain optimal athletic performance on a low energy intake. Thus, advice given to athletes should include not skipping meals and daily intakes of healthy snacks. This emphasizes the need for development and collaboration of sports nutritionist/dietitians with athlete trainers/coaches in our various institutions and the sports industry in Nigeria.

## 6. Limitation and Suggestions for Further Research

Depending on the type of sport, there are slight variations that exist in the need for nutrients and this study did not consider the various activities that the athletes are engaged in. Rather, the general recommendations for the nutrients were used. We assessed nutritional knowledge and practices of Undergraduates in University of Ibadan. Future research could include a broader population including elite athletes in different sports across the country. Further, intervention studies are needed to emphasize nutrition education aimed at improving athletes’ performance.
